# The Ætiology of Diphtheria

**Published:** 1878-02

**Authors:** 


THE ÆTIOLOGY OF DIPHTHERIA:

Dr. Thorne has observed and reported an extensive epidemic of diphtheria occurring at Great Coggeshall, in Essex. The outbreak commenced in November, 1875, and continued till the end of 1876. The disease was at first not recognized, the first seven deaths being registered as due to "croup." Dr. Thorne, however, satisfied himself that these early cases were diphtheria, showing amongst other things that the disease was from the start of an infectious character. No antecedent case could be heard of, and both Great Coggeshall and the surrounding district had been for some time remarkably free from those diseases liable to affect the throat, thus having more or less affinity with diphtheria. There were no diseases among the cows that might affect the milk supply, nor were there any known sanitary defects common to the houses first attacked. Dr. Thorne confidently expresses the opinion that the first case or cases had not been arrived at. Strong evidence is brought to show the infectious nature of the disease; in one series of cases the infection is traced through fifteen persons, starting from two children who were attending school. The whole place is remarkable for the dampness of its site. The effect of temperature and rainfall on the spread of the disease was very marked, and from the tables Dr. Thorne has prepared it is seen that the largest number of attacks occurred during the months of excessive rainfall and comparatively low temperature, whereas, with a rising thermometer and a diminished rainfall, the number of attacks became smaller. Owing to the bad sanitary arrangements throughout the entire place, observations on this point were unsatisfactory, but it was noticeable nevertheless, that a very large proportion of the cases occurred in overcrowded and ill-ventilated houses. In two instances second attacks occurred, and in each the second attack was far worse than the first. In one of them the last attack was fatal.—*The Practitioner,* November.

